# Pre-eclampsia with severe features: management of antihypertensive therapy in the postpartum period

**DOI:** 10.11604/pamj.2020.36.216.19895

**Published:** 2020-07-27

**Authors:** Nnabuike Chibuoke Ngene, Jagidesa Moodley

**Affiliations:** 1 Department of Obstetrics and Gynaecology, University of KwaZulu-Natal, Durban, South Africa,; 2 Department of Obstetrics and Gynaecology, Klerksdorp Hospital, North West Province, Klerksdorp, South Africa,; 3 Department of Obstetrics and Gynaecology, School of Clinical Medicine, Faculty of Health Sciences, University of the Witwatersrand, Johannesburg, South Africa

**Keywords:** Alpha-methyldopa, antihypertensive drug combination therapy, amlodipine, postpartum hypertension, severe preeclampsia

## Abstract

**Introduction:**

there is variance in both the types and combinations of antihypertensive drugs used for managing pre-eclampsia in the postpartum period. Knowledge of the most common and suitable single or combination antihypertensive drug therapies in the postpartum period will minimize harmful effects, promote adherence to medications, overcome any fears that lactating mothers may have about these drugs and will assist in healthcare planning. Objective: to determine the types of antihypertensive drug therapies used in managing pre-eclampsia with severe features (sPE) in the postpartum period in a regional hospital in South Africa.

**Methods:**

fifty consecutively presenting pregnant women with sPE were followed up prospectively from the pre-delivery period (within 48 hours before delivery) until day 3 postpartum. The antihypertensive drug therapies administered to the participants were observed. Their blood pressures were measured daily at 04: 00, 08: 00, 14: 00 and 22: 00 hours.

**Results:**

nifedipine was the commonest rapid-acting agent used for severe hypertension. Prepartum, 9 different combinations of antihypertensive drugs were prescribed; alpha-methyldopa was the commonest single long-acting agent used. Postpartum, the number of different drug combinations administered were 15, 18, 22 and 16 on days 0, 1, 2 and 3 respectively. Alpha-methyldopa was the commonest single agent used on postpartum days 0 - 2 while hydrochlorothiazide was the most frequently used single agent on postpartum day 3. Postpartum, the commonest combination therapy was alpha-methyldopa and amlodipine on day 0; alpha-methyldopa and amlodipine as a regimen as well as alpha-methyldopa, amlodipine and hydrochlorothiazide as another regimen on day 1; alpha-methyldopa and amlodipine on day 2; and many amlodipine-based regimens on day 3.

**Conclusion:**

a variety of antihypertensive drug combinations were used in the postpartum period indicating the need for standardised guidelines; however, detailed studies are required to evaluate their efficacies completely.

## Introduction

Pre-eclampsia (PE) affects 4.6% (95% uncertainty range 2.7-8.2) of pregnancies globally and constitutes a major cause of adverse pregnancy outcomes [1]. Of the global 830 maternal deaths that occurred daily in 2015, hypertensive disorders of pregnancy (HDP) particularly PE accounted for 14% of the mortalities [2]. A substantial proportion of deaths occurred due to poorly managed hypertension in women who had PE with severe features (sPE) that developed cerebral haemorrhage and or eclampsia [3]. To prevent maternal mortality and morbidity from severe hypertension, the judicious use of rapid-acting antihypertensive agents is required. Obstetrician´s familiarity with an antihypertensive drug is a key factor that guides the selection of medication in the postpartum period [4]. Other factors include drug availability and the patient´s clinical condition. Another important consideration is pregnancy physiology such as increased plasma volume and decreased protein binding which may affect drug distribution [5,6]. In the immediate postpartum period, blood pressure (BP) levels typically increase [7], and the rises in BP is attributable to clinical practices (such as fluid therapy) and physiological changes (including mobilization of interstitial oedema fluid and shift of blood volume from the uterine to the extra-uterine circulation) [6-8].

In PE without severe features, the value of antihypertensive therapy is controversial [9], but recent data suggest that it may prevent further increases in BP levels without any recognizable harm [10]. Treatment of mild chronic hypertension in pregnancy has similarly been reported to be beneficial [10,11] although the American College of Obstetricians and Gynaecologists recommends less restrictive BP targets (systolic 120 to <160 mmHg and diastolic 80 to <110 mmHg) [12] thus suggesting doubt about the need for a tight BP control. Before these findings, antihypertensive drug therapy in PE without severe features was thought to reduce fetal perfusion [13] but may be prescribed based on indication [13,14]. Studies in the prenatal period have improved the use of antenatal antihypertensive agents [15] and in many countries especially in low- and middle-income countries, the first-line antihypertensive drug during this period is alpha-methyldopa [16]. In the postpartum period, only limited data are available to guide the drug management of hypertension [17-19]. Although the effects of medication on lactation is a concern for clinicians and patients, a wide range of antihypertensive agents may be considered postpartum unlike in the antenatal period when the fetus is at risk of teratogenicity in the first trimester.

The availability of a wide range of antihypertensive drugs results in variance on the type and sequence of selection of antihypertensive agents in the postpartum period. An unjustifiable variation in drug treatment adjustment, as reported by some investigators, [20] is therefore not an uncommon postpartum observation. Unfortunately, there is limited data on the efficacy of these various combinations of antihypertensive agents used postpartum [5,21] particularly in South Africa (SA). Investigators, therefore, continue to recommend management strategies [22,23] and monitoring of drug therapy in HDP even in the puerperium [21] given that severe hypertension and eclampsia are not uncommon in the postpartum period. Knowledge of the various antihypertensive drug combinations used in pregnancy and postpartum period will assist in healthcare planning and in counselling of patients about the treatment that they are likely to receive. In this study, therefore, we aimed to determine the types of antihypertensive drugs (single and combination therapies) utilized in the postpartum period in a regional hospital in SA to manage women with sPE that had abdominal deliveries. The daily mean BPs associated with the use of these antihypertensive drugs were also assessed.

## Methods

**Type of study:** this was a prospective observational study.

**Site and regulatory permission:** this study was conducted between August and December 2015 in a regional hospital in South Africa. Before the study, ethical approval was granted by the Biomedical Research Ethics Committee (BREC) of the University of KwaZulu-Natal (reference BE236/14). Each patient gave informed consent before participation in the study.

**Participants:** the study participants were consecutively presenting women with sPE that had caesarean delivery (CD). In our setting, limited availability of hospital bed spaces [24] makes it unfeasible to increase the duration of inpatient monitoring of all women that have vaginal deliveries for research purposes. Loss to follow-up was also envisaged to be high if BPs were to be measured on an outpatient basis in postpartum women. For instance, a report from the USA indicates a 48% loss to follow-up rate among sPE [25]. Even with the use of an ambulatory BP monitor, 15% of patients will discontinue from a study due to the discomfort and the night disturbances caused by the device [26].

This study was a follow-up analysis of a study on postpartum BP and pre-delivery serum concentration of angiogenic factors in sPE and normotensive pregnant women to identify clinical usefulness of angiogenic imbalance [7,23]. Therefore, we included only sPE (and not PE without severe features) given that this group usually manifest intense angiogenic imbalance (ratio of soluble fms-like tyrosine kinase-1 [sFlt-1] to placental growth factor [PIGF]). A previous report from the base study showed that only one patient with de-novo postpartum hypertension required antihypertensive drug therapy [7] therefore, a normotensive group was excluded from the index study. Pre-eclampsia was defined as new-onset hypertension (BP ≥ 140/90 mmHg) at ≥ 20 weeks gestation with any of significant proteinuria, maternal organ dysfunction or uteroplacental insufficiency [27]. The diagnostic criteria for sPE included: systolic BP ≥ 160 mmHg, diastolic BP ≥ 110 mmHg, elevated liver transaminases twice the normal values, elevated serum creatinine twice the normal levels, HELLP syndrome, features of impending eclampsia, pulmonary oedema, thrombocytopenia < 100 X 10^9^/L, proteinuria ≥ 3 g/24 hours, and severe placental insufficiency. We reorganize the differences expressed by investigators concerning the diagnostic criteria for sPE including the recent recommendation for increased surveillance when proteinuria exceeds 2 g/24 hours in PE [28].

In the study setting, all patients with sPE were admitted in the obstetric high care unit. Experienced specialist obstetricians supervised patients´ management. The timing of delivery in sPE was indicated by gestational age <24 weeks, deterioration in maternal conditions, fetal compromise, fetal demise, inability to control BP with maximal doses of 3 antihypertensive drugs from different classes, and or attainment of 34 gestational weeks [27]. To prevent eclamptic fits, sPE women received MgSO_4_ infusion for a duration not exceeding 24 hours following childbirth. Postpartum, BP ≥ 150/100 mmHg was treated with antihypertensive drug(s) [19]. In the presence of organ failure such as renal impairment, BP ≥ 140/90 mmHg was also treated. Rapid-acting antihypertensive drug was used to control severe hypertension (BP ≥ 160/110 mmHg). Slow-acting antihypertensive drug was used to maintain the BP control. Additional antihypertensive drug was included in the regimen if the BP was uncontrolled [29].

**Data collection:** the BP of each patient was measured daily at 04: 00, 08: 00, 14: 00 and 22: 00 hours with the Mindray iMEC12 patient monitor (Shenzhen Mindray Bio-Medical Electronics Co., Ltd.), a device which has been validated for use in pregnancy [30]. Standard technique [31] was used by experienced nurses to measure the BPs. Details of antihypertensive drug therapies were extracted daily from each patient´s hospital chart by a trained research assistant. Following childbirth, the observation period was from postpartum day 0 - 3. The day of childbirth was regarded as day 0 postpartum. A day of hospital stay was defined as the period from 00: 00 hours to the next 23: 59 hours.

**Data analysis:** statistical analysis of data was performed using SPSS version 25.0 (IBM, Armonk, NY, USA). Categorical variables are presented as frequencies and percentages. Continuous but normally distributed data are presented as mean with standard deviations.

## Results

A single criterion was used to diagnose sPE in 21/50 (52%), of which 20/50 (40%) had severe hypertension while 1/50 (2%) had impending eclampsia. Most patients (29/50) had multiple features of sPE with the commonest being impending eclampsia with severe hypertension 11/50 (22%) while the second commonest multiple features were severe hypertension with proteinuria 5/50 (10%). In both those with single and multiple features, severe hypertension occurred in 44/50 (88%) while impending eclampsia occurred in 21/50 (42%). The majority (45/50) of the patients had spinal anaesthesia using hyperbaric bupivacaine while the rest had general anaesthesia. Table 1 shows the clinical characteristics of the patients and perinatal outcomes. A total of 49/50 (98%) and 48/50 (96%) of the patients were on antihypertensive drug therapy in the pre-delivery and postpartum (days 0 - 3) periods respectively. Table 2, Table 3, Table 3 (suite) show the different types of antihypertensive drugs that were administered during the pre-delivery period and on postpartum days 0 - 3. Pre-delivery, the mean daily BP of users of different antihypertensive drugs ranged from 104 - 183 systolic and 66 - 106 mmHg diastolic (Table 2). On postpartum days 0, 1, 2 and 3, the daily mean systolic BPs ranged from 117 - 175, 116 - 153.75, 121.75 - 176 and 137.25 - 160 mmHg respectively among users of different antihypertensive drug therapy (Table 3, Table 3 (suite)). During the same period, the daily mean diastolic BPs were 69.50 - 96, 66.75 - 95.50, 69.75 - 102 and 76.63 - 110 mmHg on postpartum days 0, 1, 2 and 3 respectively (Table 3, Table 3 (suite)).

**Table 1:** maternal characteristics and fetal outcomes

Variable	Value in frequency (%) or mean ± SD
**Age (years)**	
≤19	15(30)
20 - 34	30(60)
35-40	5(10)
≥41	0
**Parity**	
Primigravida	23 (46)
Multigravida	27(54)
**Body mass index (kg/m^2^) before delivery**	32.22 ± 6.55
**Gestational age at diagnosis of preeclampsia**	
<34 weeks	17(34)
≥34weeks	33(66)
**Gestational age (weeks) at delivery**	
<34	17(34)
34 - 36	11(22)
≥37	22(44)
**APGAR score of the baby in 5 minutes**	
0	4(8)
1 - 6	5(10)
≥7	41(82)
**Birth weight of the baby (kg)**	
< 1.0 (extremely low birth weight)	9(18)
1 - <1.5 (very low birth weight)	4(8)
1.5 - 2.49	18(36)
2.5 - 3.99	18(36)
≥ 4.0	1(2)

**Table 2:** predelivery antihypertensive drugs and blood pressures before premedication for delivery

Antihypertensive drugs and number of users, n	Blood pressure (mmHg)	
	Systolic BP	Diastolic BP
None n = 1	161.00 ± 0	104.00 ± 0
Methyldopa, n = 29	141.00 ± 15.60	90.18 ± 11.22
Methyldopa, Nifedipine XL, n = 2	150.00 ± 2.83	106.50 ± 13.44
Methyldopa, Monohydrallazine, n = 4	136.67 ± 19.04	87.00 ± 16.37
Methyldopa, Monohydrallazine, Nifedipine XL, n = 1	104.00 ± 0	66.00 ± 0
Labetalol iv, Methyldopa, n = 1	183.00 ± 0	113.00 ± 0
Methyldopa, Nifedipine capsule, Nifedipine XL, n = 1	180.00 ± 0	102.00 ± 0
Methyldopa, Nifedipine capsule, n = 9	141.89 ± 16.27	94.22 ± 10.17
Amlodipine, Methyldopa, n = 1	149.00 ± 0	94.00 ± 0
Labetalol iv, Methyldopa, Nifedipine capsule, n = 1	150.00 ± 0	102.00 ± 0

Abbreviations: BP, Blood pressure; iv, intravenous; Methyldopa, alpha-methyldopa; Nifedipine capsule, rapid-acting nifedipine; Nifedipine XL, slow-release nifedipine; none, no antihypertensive drug required or administered.

**Table 3:** postpartum antihypertensive drugs and the daily mean blood pressures among users

Day 0 postpartum					Day 1 postpartum				Day 2 postpartum					Day 3 postpartum			
Antihypertensive drugs and number of users	BP				Antihypertensive drugs and number of users	BP			Antihypertensive drugs and number of users		BP			Antihypertensive drugs and number of users	BP		
	SBP		DBP			SBP		DBP			SBP		DBP		SBP	DBP	
None, n = 2	110.0 ±0		64.0 ±0		None, n = 9	131.61 ±17.53		79.88 ±10.74	None, n = 11		140.63 ±7.95		87.90 ±6.61	None, n = 18	141.49 ±15.86	89.34 ±10.75	
Methyldopa, n = 24	133.91 ±13.97		81.12 ±11.15		Methyldopa, n = 12	131.52 ±11.05		81.49 ±8.35	Methyldopa, n = 8		133.41 ±15.78		81.29 ±13.90	Methyldopa, n = 3	138.17 ±2.84	87.67 ±9.57	
Methyldopa, hydrallazine, Nifedipine capsule, n = 1	175.00 ±0		96.00 ±0		Amlodipine, Hydrallazine, Methyldopa, n = 2	137.88 ±4.07		88.50 ±7.07	Hydrallazine, Nifedipine capsule, n = 1		147.00 ±0		90.00 ±0	Amlodipine, Enalapril, Hydrallazine, n = 1	160.00 ±0	110.0 ±0	
Nifedipine XL, n = 1	146.00 ±0		83.00 ±0		HCT, Methyldopa, Nifedipine XL, n = 1	142.00 ±0		72.75 ±0	HCT, Nifedipine capsule, n = 1		156.50 ±0		93.00 ±0	Amlodipine, Enalapril, HCT, n = 2	151.25 ±13.44	96.13 ±6.89	
Amlodipine, HCT, Methyldopa, n = 2	146.30 ±3.54		93.00 ±5.66		Enalapril, n = 1	151.75 ±0		95.25 ±0	Amlodipine, Enalapril, HCT, Hydrallazine, n = 1		176.00 ±0		102.00 ±0	HCT, n = 4	144.33 ±10.96	92.08 ±0.58	
Methyldopa, Nifedipine capsule, n = 2	137.25 ±1.77		79.75 ±8.84		Amlodipine, n = 5	134.50 ±8.77		81.85 ±10.01	Amlodipine, n = 5		145.70 ±7.30		86.65 ±16.54	Amlodipine, Hydrallazine, n = 1	151.67 ±0	94.00 ±0	
Amlodipine, HCT, n = 1	130.50 ±0		82.00 ±0		Amlodipine, HCT, Methyldopa, n = 3	121.58 ±14.47		80.50 ±11.72	HCT, Methyldopa, n = 1		128.75 ±0		78.00 ±0	Amlodipine, Nifedipine capsule, n = 2	143.63 ±1.24	93.38 ±1.94	
HCT, Methyldopa, Hydrallazine, n = 1	122.00 ±0		68.50 ±0		Amlodipine, Enalapril, Methyldopa, n = 1	129.75 ±0		77.75 ±0	Amlodipine, Enalapril, HCT, n = 1		147.75 ±0		88.00 ±0	Amlodipine, n = 2	147.75 ±15.91	92.75 ±4.60	
Amlodipine, n = 3	135.00 ±21.21		75.50 ±2.12		HCT, Hydrallazine, Methyldopa, n = 1	116.00 ±0		67.75 ±0	Amlodipine, Enalapril, Methyldopa, n = 1		165.00 ±0		103.00 ±0	Amlodipine, Enalapril, Nifedipine capsule, n = 1	154.75 ±0	84.50 ±0	
Amlodipine, Methyldopa, n = 5	134.85 ±3.68		78.35 ±8.12		Amlodipine, Enalapril, HCT, n = 2	124.67 ±21.68		80.46 ±12.43	HCT, Methyldopa, Hydrallazine, n = 1		121.50 ±0		74.50 ±0	Enalapril, HCT, n = 1	131.00 ±0	81.50 ±0	
HCT, n = 1	117.00 ±0		69.50 ±0		HCT, Nifedipine capsule, n = 1	153.75 ±0		90.50 ±0	Amlodipine, Hydrallazine, n = 1		140.00 ±0		77.25 ±0	Enalapril, HCT, Methyldopa, n = 2	145.13 ±4.07	94.25 ±6.01	
Enalapril, HCT, Methyldopa, n = 1		130.00 ±0	88.67 ±0	Amlodipine, Enalapril, n = 2		120.0 ±21.21	74.00 ±16.26			Amlodipine, Nifedipine capsule, n = 1	152.25 ±0	95.25 ±0		Amlodipine, HCT, Methyldopa, n = 1	156.00 ±0	101.00 ±0
Enalapril, HCT, n = 1		160.00 ±0	89.50 ±0	HCT, n = 1		146.75 ±0	95.50 ±0			Amlodipine, Enalapril, n = 2	121.75 ±12.37	69.75 ±13.44		Amlodipine, HCT, Hydrallazine, Methyldopa, n = 1	137.25 ±0	98.25 ±0
Methyldopa, hydrallazine, Labetalol iv, n= 1		143.00 ±0	93.00 ±0	Methyldopa, Nifedipine capsule, n = 2		140.00 ±11.67	87.88 ±0.89			HCT, n = 2	131.13 ±14.32	85.50 ±11.31		Amlodipine, Methyldopa, n = 2	138.75 ±2.83	89.50 ±0
Enalapril, Furosemide, n = 1		Missing Data	Missing Data	Amlodipine, Nifedipine capsule, n = 1		150.00 ±0	94.75 ±0			Enalapril, HCT, n = 1	129.75 ±0	86.00 ±0		Amlodipine, Enalapril, n = 2	142.25 ±25.81	90.16 ±16.97
Methyldopa, Hydrallazine, n = 1		124.00 ±0	78.00 ±0	Enalapril, HCT, Methyldopa, n = 1		133.33 ±0	74.00 ±0			Enalapril, n = 1	150.50 ±0	93.00 ±00		Amlodipine, HCT, n = 1	139.33 ±0	91.00 ±0
				Amlodipine, Methyldopa, n = 3		125.78 ±4.13	81.03 ±4.14			Amlodipine, HCT, Methyldopa, n = 1	143.33 ±0	93.33 ±0		Enalapril, n = 2	138.63 ±16.09	76.63 ±9.02
				HCT, Hydrallazine, Labetalol iv, Methyldopa, n = 1		140.00 ±0	87.25 ±0			Enalapril, HCT, Methyldopa, n = 3	144.72 ±9.90	87.67 ±5.44		Missing Data, n = 4	142.08 ±15.20	91.58 ±14.68
				Enalapril, Furosemide, n = 1		130.75 ±0	66.75 ±0			Amlodipine, Methyldopa, n = 3	132.94 ±16.88	80.33 ±12.19				
										Amlodipine, HCT, n = 1	143.50 ±0	88.25 ±0				
										Enalapril, HCT, Hydrallazine, Labetalol iv, Methyldopa, n = 1	151.75 ±0	99.00 ±0				
										Enalapril, Furosemide, n = 1	139.25 ±0	73.75 ±0				
										Methyldopa, Enalapril, n = 1	138.75 ±0	85.50 ±0				

Abbreviations: BP, Blood pressure; DBP, diastolic blood pressure; hydralazine, monohydrallazine (oral hydrallazine); HCT, hydrochlorothiazide; iv, intravenous; Methyldopa, alpha-methyldopa; Nifedipine capsule, rapid-acting nifedipine; Nifedipine XL, slow-release nifedipine; none, no antihypertensive drug required or administered; SBP, systolic blood pressure.

## Discussion

**Main findings:** as many as 9 - 22 different antihypertensive regimens were used in the pre-delivery and postpartum periods. In the pre-delivery and on days 0 - 2 postpartum, alpha-methyldopa was the commonest used single agent. Hydrochlorothiazide was singly the most used on postpartum day 3. On postpartum days 0 - 3, amlodipine-based regimens were the commonest used combination therapy. The commonest rapid-acting antihypertensive agent used in the pre-delivery and postpartum period was nifedipine. Additionally, the initiation of antihypertensive drug therapy did not always result in normalization of the BP.

**Strengths and weaknesses:** the definition of a day of hospital stay from 00: 00 midnight to 23: 59 hours could have affected the number of BP recordings utilized to calculate the mean BP. For instance, patients that were admitted to the hospital or who developed severe hypertension close to 23: 59 hours had fewer numbers of BP recordings than those admitted earlier the same day. In tandem, the effect of a therapy administered approximately at 23: 59 hours were probably observed the next day. In this study, therefore, the daily mean BP may not necessarily reflect the effects of the daily antihypertensive drug therapy. Expressed in another way, it is difficult to adjust for the persistence of the BP-lowering effect of the preceding drug therapy. Therefore, future studies assessing drug effectiveness are required. Although challenging, the studies should be designed to calculate the blood concentration (i.e. normal, below or above the therapeutic level) of the proceeding antihypertensive drug(s) as well as the BP decrease that occurs following commencement of a new drug therapy. However, the effect of vasoactive medications such as MgSO_4_ administered to women with sPE may affect the BP.

The inclusion of only women who had CD is another limitation of this study. Caesarean delivery performed under spinal anaesthesia is associated with hypotension predominantly by sympathetic blockade that reduces vascular tone preferentially in the arteries than veins [32]. Aortocaval compression in addition to the rapid onset of the sympathetic blockade may worsen the hypotension [33]. The frequent pre-loading and co-loading with intravenous fluid as well as the incessant use of inotropes due to hypotension during CD improve the BP. Nonetheless, the duration of action of hyperbaric bupivacaine is limited to between 90 and 200 minutes [34]. Notably, a study involving 30 sPE and 30 healthy pregnancies had shown that sPE women suffer less hypotension than normotensive women during spinal anaesthesia for elective CD [35]. Other experts share the same view and equally support restrictive fluid therapy as fluid pre-loading disrupts endothelial glycocalyx [36]. Another weakness of our study is the limited number of the sample size which constrained the number of patients using different antihypertensive drug therapy. Future studies on this topic should address this weakness. Nonetheless, a number of strengths are demonstrated by this study. There is still limited data on antihypertensive drug combinations administered to patients with sPE. To the best of our knowledge, this is the first time a detailed prospective data on antihypertensive drugs (single and combination therapy) used in a regional hospital in the African continent for sPE has been profiled and reported. This study fills this knowledge gap and may act as a spur to future research. Additionally, the findings of this study will be useful to healthcare managers and clinicians to plan drug procurement and patient counselling.

**Interpretation:** the more the number of antihypertensive drugs administered, the higher the BP was and or difficult to control in that user. Contact with a healthcare facility and initiation of antihypertensive drug therapy does not always result in normalization of the BP as shown in Table 2, Table 3, Table 3 (suite). Therefore, women with sPE should be managed in an obstetric high care unit or a bed dedicated for these purposes to ensure frequent monitoring and adjustment of drug therapy.

Alpha methyldopa was most commonly used antenatally. This is related to its long-standing history of safety profile in pregnancy [16]. This prescription is in agreement with current guidelines [27]. The recommendation to taper the dose of apha-methyldopa and switch to another agent is a contributor to continued use of the drug up to postpartum day 3. If there is renal impairment, some clinicians prefer to continue the use of alpha-methyldopa. In the absence of a compelling indication, it is recommended that alpha-methyldopa be withdrawn in the first 48 hours following childbirth [19] because of the associated risk of postpartum depression. Amlodipine was the second commonest slow-acting antihypertensive drug administered postpartum possibly due to its once-daily dosing and renal friendly nature. Approximately 17.6% of women in SA with PE were reported to have kidney injury [37], and it may be reasonable to use agents that have the least effect on renal function. This may account for amlodipine being the commonest agent contained in the combined antihypertensive drug therapy used during the postpartum period. Angiotensin converting enzyme inhibitors (ACEI) such as enalapril interfere with renal function as they preferentially dilate efferent renal arterioles decreasing glomerular filtration rate. Therefore, ACEI should be used with caution in patients with renal impairment. Understandably, some clinicians prefer ACEI as the first-line agent in the postpartum period. Furthermore, the use of diuretics in the postpartum period may interfere with lactation but may be of value in patients with pulmonary oedema [38] and those with “difficult-to-control” BP. Additionally, beta-blockers such as atenolol may be used in the postpartum period but were not administered to any of the patients during the study possibly because the majority of them were young Black Africans, who are reported to have elevated renin levels that may affect their response to this agent [39]. In the authors´ experiences, beta-blockers are valuable although they may be inferior to other agents such as calcium channel blockers and diuretics in reducing stroke, renal failure and major cardiovascular disease events [40]. Rapid-acting nifedipine was the commonest administered rapid-acting agent probably because of its ease of administration and availability although it may cause and or worsens tachycardia. Recent guidelines recommend the use of rapid-acting nifedipine, labetalol or dihydrallazine as the first-line agent for the treatment of severe hypertension [19]. Notably, recent reviews [3,29,41,42] contain the essential clinical pharmacology of the drugs used for the treatment of HDP.

Unfortunately, there is still limited data to guide postpartum antihypertensive drug therapy [17]. Nonetheless, it is important to emphasize that stroke is three times commoner in pregnant than non-pregnant women [43] and 14.2% of postpartum stroke is associated with HDP [44]. Control of hypertension in PE is therefore of critical importance [44]. Another consideration includes that with or without PE, severe hypertension (controlled or uncontrolled) is an independent risk marker for adverse pregnancy outcomes [45]. Therefore, the key is to prevent the development of severe hypertension through prevention of PE with the use of agents such as aspirin and calcium supplementation in high-risk women [46] and also ensure treatment of hypertension. Notably, tight BP control results in good patient satisfaction [47].

**Recommendations on postpartum antihypertensive drug therapy:** following childbirth, the dose of alpha-methyldopa should be tapered and finally withdrawn. If the BP levels remain high, another antihypertensive drug should be introduced to replace the alpha-methyldopa. Clinicians should consider using a calcium channel blocker such as amlodipine or an ACEI inhibitor as the first (additional) antihypertensive medication to be administered in the postpartum period in a breastfeeding mother. Calcium channel blocker is preferable in women of Black African or Caribbean descents [19]. If there is renal impairment, a calcium channel blocker may be preferable to AECI. If there is tachycardia, calcium channel block will not be preferable. Other antihypertensive agents may be added to the regimen based on availability, cost considerations, patient´s clinical condition and clinician´s familiarity with an antihypertensive agent (including their contraindications, compelling indication and side-effects). Diuretics may interfere with lactation but should be used if the patient is not breastfeeding and particularly in the presence of a compelling indication such as pulmonary oedema; therefore, judicious fluid therapy is crucial. Furosemide is not an antihypertensive drug but it may be used to potentiate the effects of other antihypertensive drugs and reduce the need for an additional agent [48]. Of note, severe hypertension should be managed as an emergency using rapid-acting agents such as intravenous labetalol, rapid-acting nifedipine and dihydralazine, and evidence-based dynamic algorithms are available to guide their use [41]. If severe hypertension becomes complicated by pulmonary oedema, a nitrate such as nitroglycerin is the treatment of choice [49]. Furthermore, there is a need to “stabilize” high BP levels before hospital discharge. Given that recent evidence shows that BP increases from postpartum day 1 in both normotensive and sPE [7], it is preferable to provide inpatient care for at least 3 days and a follow-up monitoring on postpartum days 6 and 42 or more frequently depending on the clinical conditions.

Institutional clinical protocols should be available to guide practice. However, pragmatic management as determined by the clinical condition will improve patient-centred care. In “difficult-to-manage” cases, an internal medicine specialist (internist) should be consulted and multidisciplinary team management is strongly recommended. Involving an internal medicine specialist is of particular importance given the strong evidence that women with PE are likely in the long-term to develop cardiovascular disorders and the metabolic syndrome. Such patients therefore require follow-up visits to an internal medicine specialist at least once a year. Understandably, the inadequate obstetrical practice exposure of some non-obstetric experts is a major concern [50].

## Conclusion

Myriad of antihypertensive drug combinations are used to manage sPE. The knowledge of these medications will aid informative planning which is key to improving the health care system. However, additional studies on drug efficacy are required to further guide the choice of therapy for sPE in the postpartum period.

### What is known about this topic

- Poorly managed pregnancy hypertension is a major cause of maternal mortality;
- The availability of a wide range of antihypertensive drugs in the postpartum period results in variance on the type and sequence of selection of antihypertensive drug regimen among clinicians;
- There is an unjustifiable variation in antihypertensive drug treatment adjustment in the postpartum period when managing pre-eclampsia.

### What this study adds

- The commonest antihypertensive drugs used in the immediate postpartum period in sPE in the study setting are alpha-methyldopa, nifedipine, hydrochlorothiazide and various amlodipine-based combination therapies;
- Rapid-acting nifedipine was the commonest administered rapid-acting agent probably because of its ease of administration and availability;
- Women with sPE should be managed in an obstetric high care unit because antihypertensive drug therapy does not always result in normalization of the blood pressure.
